# The full-body illusion changes visual depth perception

**DOI:** 10.1038/s41598-023-37715-8

**Published:** 2023-06-29

**Authors:** Manuel Bayer, Sophie Betka, Bruno Herbelin, Olaf Blanke, Eckart Zimmermann

**Affiliations:** 1 Department of Experimental Psychology, Heinrich-Heine-University, Düsseldorf, Germany; 2 Laboratory of Cognitive Neuroscience, NeuroX Institute & Brain Mind Institute, Ecole Polytechnique Fédérale de Lausanne (EPFL), Geneva, Switzerland; 3 Department of Clinical Neuroscience, Geneva University Hospital, Geneva, Switzerland

**Keywords:** Psychology, Human behaviour

## Abstract

Knowing where objects are relative to us implies knowing where we are relative to the external world. Here, we investigated whether space perception can be influenced by an experimentally induced change in perceived self-location. To dissociate real and apparent body positions, we used the full-body illusion. In this illusion, participants see a distant avatar being stroked in virtual reality while their own physical back is simultaneously stroked. After experiencing the discrepancy between the seen and the felt location of the stroking, participants report a forward drift in self-location toward the avatar. We wondered whether this illusion-induced forward drift in self-location would affect where we perceive objects in depth. We applied a psychometric measurement in which participants compared the position of a probe against a reference sphere in a two-alternative forced choice task. We found a significant improvement in task performance for the right visual field, indicated by lower just-noticeable differences, i.e., participants were better at judging the differences of the two spheres in depth. Our results suggest that the full-body illusion is able to facilitate depth perception at least unilaterally, implying that depth perception is influenced by perceived self-location.

## Introduction

Our perception of depth is constructed from monocular and binocular cues with a preponderance of the latter in natural vision^1,2^. Retrieving spatial information from binocular disparity, i.e., the difference in the retinal projection of objects on the left and right eye, is inherently ambiguous^3,4^. A small but close object can generate the same retinal projection as a bigger object located far away. In order to resolve this ambiguity, the brain might rely on sensorimotor knowledge like the distance of locomotion required to reach an object or the size of an arm movement that would be necessary to grasp the object^5,6^. The coordinates of a movement can be used to interpret the visuospatial location of the corresponding object^7^. If spatial perception is calibrated by action, then the perceived location of objects should also depend on our perceived location in space.

A convenient experimental tool to dissociate between the subjective feeling of where we are and the physical location of our body is provided by the full-body illusion^8,9^. The illusion can be created by presenting a video stream in a head-mounted display, which shows the participants’ back being stroked by the experimenter, while the physical back of the participants is also stroked synchronously^9^. The discrepancy between the visual and the tactile location of stroking produces a shift in the perceived self-location of the observer, as if participants were standing in front of their physical body. In other versions of the full-body illusion, instead of the participants’ back, an avatar is shown^10^. This induction of the full-body illusion is then usually compared to an asynchronous condition with a delay between the felt and visual stroking^9^. The delay between the visual stroking on the avatar’s back and the felt stroking on the participant’s back should impede the induction of the full-body illusion while still providing an identical visual stimulus.

Full-body illusions are descendants of the previously discovered rubber hand illusion^11^. Both illusions are driven by a conflict between senses, forcing the brain to adopt a compromise between the diverging information sources. The resulting perception follows the principles of multisensory integration, according to which the more uncertain sense is biased toward the more certain one^12^. In full-body illusions three senses are involved, including vision, proprioception and touch^13^. Since the variance in somatosensory signals is higher than in vision, participants feel like standing where they see the avatar in the full-body illusion, thus shifting self-location from an embodied self-location (cantered on the upper body; trunk and/or face, as tested without the full body illusion^14^) towards the position of the avatar^9,10,15–17^. Multisensory integration during the full-body illusion might be implemented by trimodal neurons, which are activated by the combined presence of three sensory signals^13,18,19^. Indeed, imaging studies have shown that full-body illusions involve bilateral premotor cortex, intraparietal sulcus and sensorimotor cortex^10,13,15^.

Furthermore, electrophysiological studies found that PMC and IPS host tri- and bimodal neurons with visual and somatosensory receptive fields in the arms and the trunk (for a review, see^13^). These trunk-centered receptive fields cover the whole body^20^ and are therefore well suited to bring forth the full-body illusion. Illusory self-identification with a virtual body is also associated with physiological and nociceptive changes; for instance, the skin conductance responds to a threat directed towards the virtual body^21^. The changes in touch, pain perception and physiology that occur during illusory self-identification indicate that states of illusory self-identification alter the way humans process stimuli from their body^22–24^.

Full-body illusions can be estimated by subjective measures, i.e., questionnaires, and by objective measures^17,25,26^. In questionnaires, participants report that they locate themselves closer to or at the position of the visual body or avatar. Several objective measures have provided evidence that, after the induction of the full-body illusion, participants estimate themselves to be at a different location in space than their physical body^26,27^. This shift of the perceived self-location was measured by changes in behavior or by perceptual effects which reflect the felt position of the body. An instance of an objective measure is a mental imagery task (i.e., mental ball dropping task). Lenggenhager, Mouthon and Blanke^28^ instructed their participants to imagine dropping a ball and to indicate when they think it reached the ground while being in a prone position on a bench. The estimated time before participants indicated that the ball reached the ground was shorter after the induction of the full-body illusion. In a comparable study in which a ball is approaching the participant, Nakul et al.^26^ demonstrated that participants judged the arrival of the ball earlier after the induction of the full-body illusion, compatible with a forward drift in self-location. Several other studies used blind walking tasks, during which participants were displaced and had to walk back to their initial position. Under the full-body illusion participants tended to overshoot their position, consistent with the experience of being displaced forward in space^9,10,17^.

Another approach to quantify the effects of the full-body illusion is the measurement of the shift of the peripersonal space, i.e., the space in which we integrate multisensory body-related signals around the body^29^. For instance, Noel et al.^29^ presented a looming sound and a tactile vibration to the participants, who were asked to respond as soon as they perceive the tactile stimulation. This procedure enables the measurement of the boundaries of the peripersonal space by presenting stimuli in the front and the back and makes it possible to measure the peripersonal space shifts at several distances between the real physical body of the participant and the full-body illusion related avatar.

In order to investigate if the experimentally induced drift in self-location during the full-body illusion alters depth perception we used a psychometric measurement. In this visual depth task, two spheres were presented repeatedly immediately after illusion induction. Participants were asked to judge which of the spheres was closer to them. Since depth perception is more accurate within the peripersonal space^30^ and more accurate for closer objects in general, we expected the experimentally induced self-location drift to influence the participants‘ task performance. If the peripersonal space drifts toward the avatar and therefore also toward the stimuli, participants might be able to judge the distance of the stimuli more precisely, reflected by lower just-noticeable differences (JNDs). As the task only requires the participants to judge a relative distance, we did not expect to find any effect on absolute perception, which would manifest itself in point of subjective equality (PSE) differences.

## Results

### Questionnaire

**Figure 1:**
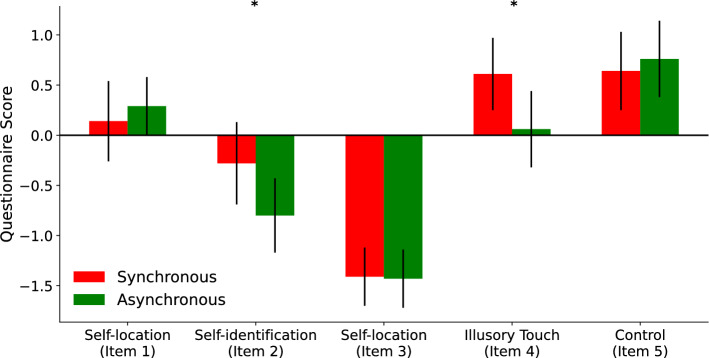
Mean scores of each full-body illusion questionnaire item for the synchronous (red) and asynchronous condition (green). Error bars indicate the standard error. There were significant differences between the two conditions for the second and fourth item. Item 2 asked the participants to what extent they felt as if the avatar they saw was their own and item 4 asked to what extent the stroking felt as if it was located on the avatar. There were no significant differences between the synchronous and asynchronous condition for the first, third and fifth item. Item 1 asked the participants to what extent they felt as if they were slightly above or below the seen avatar, item 3 asked the participants to what extent they felt as if their own body shifted towards the seen avatar and item 5 asked the participants to what extent they felt as if nothing changed. *Indicates *p*< 0.05.

Participants performed a questionnaire to quantify the intensity of the illusion. This questionnaire comprised five items, covering the following aspects of the full-body illusion: perceived self-location, self-identification and illusory touch experiences^22^. There were significant differences between the synchronous and asynchronous condition for the second (self-identification) and fourth item (illusory touch experience) of the questionnaire (item 2: *t*(19) = 2.04, *p* = 0.028, item 4: *t*(19) = 1.78, *p* = 0.045, see Fig. 1). In both cases, participants were showing more agreement to these statements in the synchronous compared to the asynchronous condition, in line with previous results^9,17,22^. This result confirms that the present setup was able to successfully induce the full-body illusion. For the other items we did not find any significant differences between the synchronous and asynchronous condition (item 1: *t*(19) = − 0.53, *p* = 0.700, item 3: *t*(19) = 0.10, *p* = 0.459, item 5 failed the test for normality, we therefore opted to perform a wilcoxon signed-rank test: *W* = 53.5, *p* = 0.475).

Based on the responses in the questionnaire we computed the average response across all items for each participant. In this calculation item 5 was mirrored, as more agreement indicated a weaker illusion. Based on this average response we split the participants in a high quotient (*M* = 1.25, *SD* = 2.41) and low quotient group (*M* = − 5.02, *SD* = 4.11) via a median split. This split was performed to account for the variance in the response to the full-body illusion. Participants with a strong full body illusion (questionnaire quotient ≥ − 1.00) were classified as high quotient and participants with a weak full body illusion (questionnaire quotient< -1.00) were classified as low quotient.

### Psychometric measures

**Figure 2:**
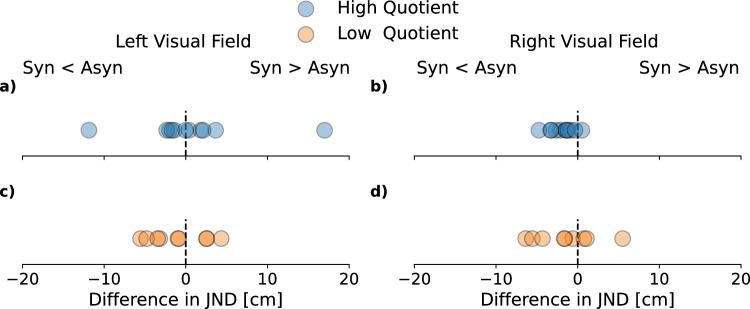
Precision of localization in the visual depth task, as quantified by JNDs. A lower JND indicates a higher sensitivity, i.e., higher precision in solving the task. Differences between the synchronous and asynchronous condition were split by the side of the visual field in which stimuli were presented (left/right) and the questionnaire group (low/high quotient). (**a**) JND differences for the high quotient group from trials in which the probe was presented in the left visual field. Data points represent single subject data. The dashed line indicates equality between the synchronous and asynchronous condition. Values to the left of the dashed line represent participants who had a lower JND in the synchronous (syn) compared to the asynchronous condition (asyn) and vice versa. (**b**) JND differences for the high quotient group from trials in which the probe was presented in the right visual field. One can see that all data points (except one) lay to the left of the dashed line, thus showing lower JNDs in the synchronous compared to the asynchronous condition. Across all participants we found a significant difference in JNDs between synchronous and asynchronous stroking sessions for the high quotient group in trials in which the probe was presented in the right visual field. (**c**) JND differences for the low quotient group in which the probe was presented in the left visual field. (**d**) JND differences for the low quotient group in which the probe was presented in the right visual field. Data are relatively evenly distributed around the dashed line, indicating no JND difference between the synchronous and asynchronous condition.

To quantify the influence of the full-body illusion on visual depth discrimination, we compared JNDs from the synchronous versus asynchronous sessions. To capture the effect of the full-body illusion, we split the participants into two groups according to the subjective strength of the full-body illusion that was estimated by the questionnaire. Figure 2b shows JND differences in cm between the synchronous and asynchronous (asynchronous–synchronous) condition for all participants of the high quotient group for trials in which the probe was presented in the right visual field. If data points lie on the dashed line, there is no difference between the two conditions. One can see that all data points (except one) lay to the left of the dashed line, thus showing lower JNDs in the synchronous than in the asynchronous sessions. Across all participants we found a significant difference in JNDs between synchronous and asynchronous stroking sessions for the high quotient group in trials in which the probe sphere was presented in the right visual field (paired *t*-test, *t*(10) = 4.16, *p* = 0.008). Figure 2a shows JNDs for all participants from sessions in which the probe was presented in the left visual field. In this condition, data points can be found on both sides of the dashed line. A paired *t*-test did not reveal a significant difference (*t*(10) = − 0.25, *p* = 0.999).

No JND differences were observed for the low quotient group. Results for the low quotient group are shown in Fig. 2c and d. Data points lie to both sides of the dashed line for trials in which the probe was presented in the left or right visual field. Paired t-tests did not reveal a significant difference, neither for the left (*t*(8) = 0.89, *p* = 0.999), nor for the right visual field (*t*(8) = 1.15, *p* = 0.999).

**Figure 3:**
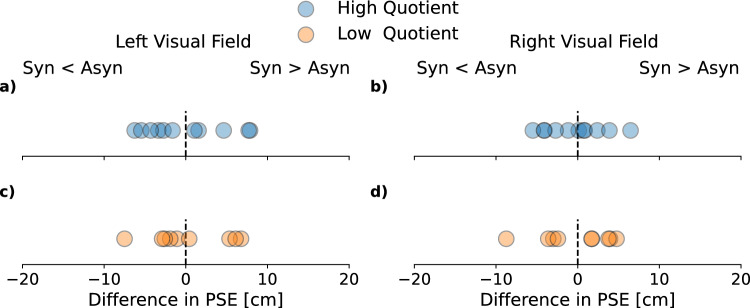
PSE differences between the synchronous and asynchronous condition split by the side of the visual field in which stimuli were presented (left/right) and the questionnaire group (low/high quotient). (**a**) PSE differences for the high quotient group from trials in which the probe was presented in the left visual field. Data points represent single subject data. The dashed line indicates equality between the synchronous and asynchronous condition. Values to the left of the dashed line represent participants who had a lower PSE in the synchronous (syn) compared to the asynchronous condition (asyn) while values to the right of the line show participants who had a higher PSE in the synchronous compared to the asynchronous condition. (**b**) PSE differences for the high quotient group from trials in which the probe was presented in the right visual field. (**c**) PSE differences for the low quotient group from trials in which the probe was presented in the left visual field. (**d**) PSE differences for the low quotient group from trials in which the probe was presented in the right visual field.

To check if the full-body illusion biased depth perception we also analyzed PSEs, by comparing the synchronous against the asynchronous condition with the same median split as for the JNDs (Fig. 3). This analysis showed no significant difference between the PSE of the high quotient group for trials in which the probe was in the left (*t*(10) = 0.05, *p* = 0.999, see Fig. 3a) or in the right visual field (*t*(10) = 0.25, *p* = 0.999, see Fig. 3b). The same was the case for the low quotient group in the left (*t*(8) = − 0.18, *p* = 0.999, see Fig. 3c) and in the right visual field (*t*(8) = 0.14, *p* = 0.999, see Fig. 3d).

These results are in line with our expectations as significant differences for the PSE would imply changes on the absolute depth perception of the participants. If the PSE of the synchronous condition was higher than the one of the asynchronous condition, participants would have perceived the probe as closer in depth after the induction of the full-body illusion.

**Figure 4:**
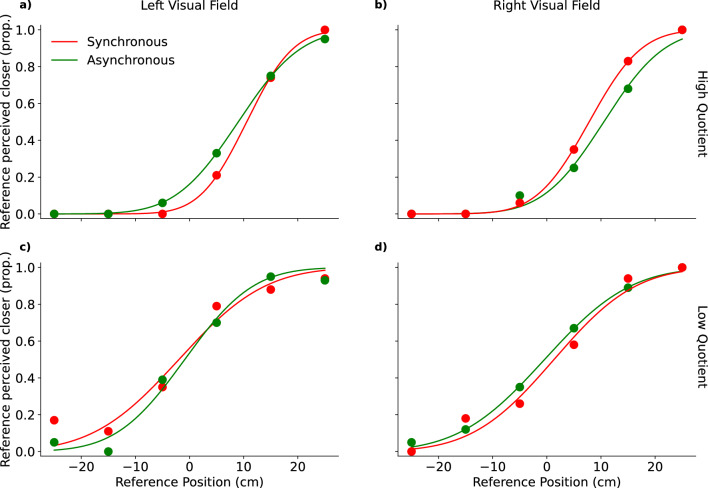
Representative psychometric functions of individual participants. (**a**) Data of a participant from the high quotient group. The ordinate indicates the proportion of cases the reference was chosen as the closer stimulus and the abscissa shows the position of the reference stimulus in relation to the probe stimulus. Higher values indicate that the reference stimulus was closer to the participant on the anterior–posterior axis and negative values indicate that the reference was further away from the participant than the probe stimulus. The lines represent the fitted cumulative gaussian functions for the synchronous (red) and asynchronous (green) condition and the dots represent the data points used for the fitting procedure. (**b**) One can see that the red curve is steeper than the green curve, indicating that this participant was more sensitive in the task after the induction of the full-body illusion in trials in which the probe was presented in the right visual field. (**c**, **d**) Little to no differences can be observed for this participant from the low questionnaire group when comparing the synchronous against the asynchronous condition for the JND.

Psychometric functions of example participants are shown in Fig. 4. Figure 4a and b represent one participant of the high quotient group and Fig. 4c and d one participant of the low quotient group. One can see that in Fig. 4b the red line is steeper than the green line, which indicates that the JND in the synchronous is lower than in the asynchronous condition in trials in which the probe was presented on the right. For the same participant this effect is less pronounced in trials in which the probe was presented on the left (see Fig. 4a). For the participant of the low quotient group there are little to no differences in steepness of the two curves for both sides of the visual field (see Fig. 4c,d).

### Mental imagery task

**Figure 5:**
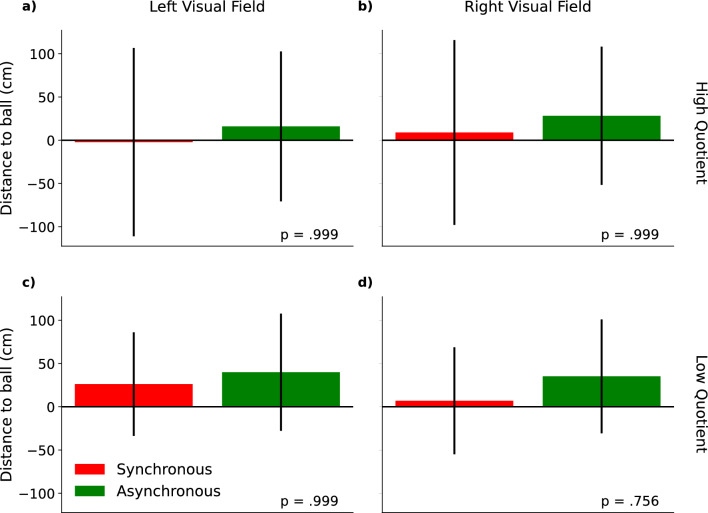
Mean distances between the ball and the participants in the mental imagery task. (**a**) The mean distances at the time of the participants’ response for the synchronous (green) and asynchronous condition (red) for the high quotient group from trials in which the ball approached the participant in the left visual field. Error bars indicate the standard error. Positive values indicate that participants responded too early, the ball was still in front of them. Negative values indicate a late response, e.g. the ball was already behind them. Participants were accurate in their judgement of the position of the approaching ball and no difference between the conditions were found. (**b**–**d**) The same pattern was observed for the high quotient group in trials in which the ball approached the participant in the right visual field and for the low quotient group irrespective of the visual field the ball was approaching from.

Based on previous data the mental imagery task was carried out to quantify changes in self-location^15,26^. We calculated the distance between the position of the participant and the ball at the time of the participant’s response. Figure 5 shows the means of all participants for the synchronous and asynchronous condition split into the high quotient and low quotient group and the side of the visual field the ball was approaching the participants from. Positive values indicate that participants responded too early, i.e. the ball was still in front of them, while negative values indicate a response that was too late, i.e. the ball had already passed their position and was behind them. We would have expected positive and higher values in the synchronous compared to the asynchronous condition if participants experienced a drift in self-location during the full-body illusion. In some cases, the mean response of the participants led to positive values (see Fig. 5c), but the differences between the individual means was small compared to the variance in the data reflected by the size of the standard error. There was no significant difference for the high quotient group between the synchronous and asynchronous condition, neither for trials in which the ball approached from the left (*t*(10) = − 0.64, *p* = 0.999, see Fig. 5a) nor in trials in which the ball approached from the right visual field (*t*(10) = − 0.55, *p* = 0.999, see Fig. 5b). The same pattern applies to the low quotient group as there was no significant difference between the synchronous and asynchronous condition independently of the visual field the ball was approaching from (left: *t*(8) = -0.63, *p* = 0.999, see Fig. 5c, right: *t*(8) = − 1.44, *p* = 0.756, see Fig. 5d).

## Discussion

We investigated whether shifts of perceived self-location can influence depth perception. To manipulate perceived self-location, we induced the full-body illusion. Participants saw an avatar being stroked on its back and synchronously felt the same stroking movement on their own physical back. The full-body illusion caused participants to perceive themselves closer to the avatar’s position due to a perceived self-location drift in the direction of the avatar^9,10,17,26,28^.

After the full-body illusion was induced, participants compared the distance of two spheres that were presented in front of them. We measured the discrimination performance after the induction and compared it to the discrimination performance in an asynchronous condition where the full-body illusion should have not been induced. The discrimination sensitivity in this task was increased when participants experienced the full-body illusion, as if they would stand closer to the discrimination targets. Except for the synchronicity of the stroking, both conditions were identical. Any improvement in discrimination can thus only be related to the synchrony of stroking that generates the multisensory conditions for the full-body illusion. Moreover, the improvement in discrimination was only present for the group of participants which experienced the full-body illusion more intensively, further providing support for a direct link between the full-body illusion and the change in depth perception.

We used objective psychometric measurements which are far less susceptible to cognitive influences than subjective measures, like verbal reports^31^. However, psychometric measures are not free of higher-level cognitive biases. It has been shown that in experiments participants can deliberately shift the bias of a psychometric function without changing its slope^32^. In our study, the slope of the function increased without concomitant change in the bias. Increasing the slope through conscious or unconscious strategies is far more unlikely than changing the bias. The latter only requires, in case of uncertainty, to respond more often with one of the two answers. The slope of the psychometric function can be changed in two directions. An individual participant can decrease the slope by simply responding more randomly. However, to increase the slope of the psychometric function, the participant must actually know the correct response. There is no reason to suppose that a group of observers can systematically guess the correct answers to pretend a signature of better discrimination performance.

How can an illusion yield better discrimination performance in depth perception? *Prima facie*, the effect might seem paradoxical, since objectively measured visual discrimination performance increases, following the induction of an illusory change in position. Improvements in discrimination through illusory changes of body-parts have been shown previously: Vignemont et al.^23^ demonstrated that the artificial elongation of individual fingers can lead to an increase in tactile discrimination sensitivity. For the present study, the projection on the two retinae produces ambiguous information about the position of objects in depth. A small but close object is connected to the same retinal image as a bigger object located far away^3,4^. Previous studies have shown that binocular depth perception is calibrated to the “natural grasping distance”, i.e. the distance that is usually chosen for manual interaction^5,6^. Planning interactions with the external world requires that we have an implicit knowledge about our own position with regard to the location of objects in the world. Put differently, executing a motor plan means to minimize the distance between start and desired end location of an effector. In order to perform a goal directed hand movement, the brain must know the current hand location that is the starting position of the hand movement.

Linkenauger et al.^33^ were able to show that we utilize the length of our limbs and the associated grasping distance to judge distances. In their study they used a virtual reality setup in which participants performed grasping movements while being represented by an avatar with various arms lengths. The mere manipulation of the avatars arm length did not have an effect on the participants’ depth perception, only after they were able to collect experiences with their manipulated arm length their judgements of distances changed. This result further provides support for the importance of grasping distance and our experiences in interacting with our environment for our perception of depth. The full-body illusion modifies the internal spatial representation of the body with regard to external space and shifts self-location towards the seen position of the avatar^9,10,17,26^. Changes in the felt position in space through the full-body illusion may affect the internal representation of the natural grasping or walking distance and thereby the calibration of visual depth.

Although we did not anticipate lateralized effects on space perception, we now discuss several factors that might contribute to it. The realism of the used avatars and the representation of the body of the participant could have had an impact on the results as well. A study of Ebrahimi et al.^34^ for example provided support for the necessity of a realistic representation of the participants body in the virtual environment in the context of depth perception. Lugrin et al.^35^ on the other hand showed that the level of anthropomorphism did not influence virtual body ownership. Participants in our study were required to give their responses with a VR controller held in their dominant hand. Since the controller followed their movement, it could have been regarded as a form of representation of their own body. As this was only the case for one side of their body, this could have impacted their depth perception unilaterally. Motor actions or the mere visual perception of an object representing their right hand might have caused recalibration processes of the peripersonal space in the respective visual field. An alternative explanation is that the full-body illusion distorts the left and right visual field unequally or only affects one. Another reason for only finding a lateralized effect might be the lack of power. While we aimed to have a sample comparable to the ones used in previous studies investigating the full-body illusion, a bigger sample size could have potentially enabled us to find the effect also for the right side of the visual field.

Our results in the mental imagery task can probably be explained by our modification of the task. The original task used by Nakul et al.^26^ used trajectories on the anterior-posterior axis while we used angled trajectories, which might have interfered with the task’s ability to quantify the full-body illusion related forward shift. The idea behind the angled trajectories was to make the task more similar to the visual depth task in which stimuli also appear in an angle relative to the participant’s view.

In conclusion, our results show that depth perception is partially calibrated by signals that mediate our perception of where we are located in space. The full-body illusion influences the interpretation of early visual processing at least unilaterally by modifying the internal representation of the body’s position in relation to external objects.

## Methods

### Participants

A total of 20 participants took part in the experiment. The sample included 13 females and seven males ($M_{age}$: 22.90, $SD_{age}$: 3.58). The sample size was determined based on the sample sizes used in similar studies. The Edinburgh Handedness Inventory was used to quantify the handedness of the participants (*M*: 77.38, *SD*: 31.32). Two participants reported that they are left handed. All participants had normal or corrected-to-normal vision. Every participant gave written informed consent prior to the experiment in accordance with the declaration of Helsinki, participated voluntarily and received either course credit or 10 € for each hour of participation as monetary compensation. This study was approved by the local ethics committee of the mathematical and natural science faculty at the Heinrich Heine University.

### Setup

After participants gave their written consent, they were equipped with a head-mounted display. The HTC Vive with Dual AMOLED 3.6” screens, a resolution of 1080 × 1200 pixels per eye (2160 × 1200 pixels combined), a refresh rate of 90 Hz and a field of view of 110 degrees was used. The participants were standing upright during the experiment.

**Figure 6:**
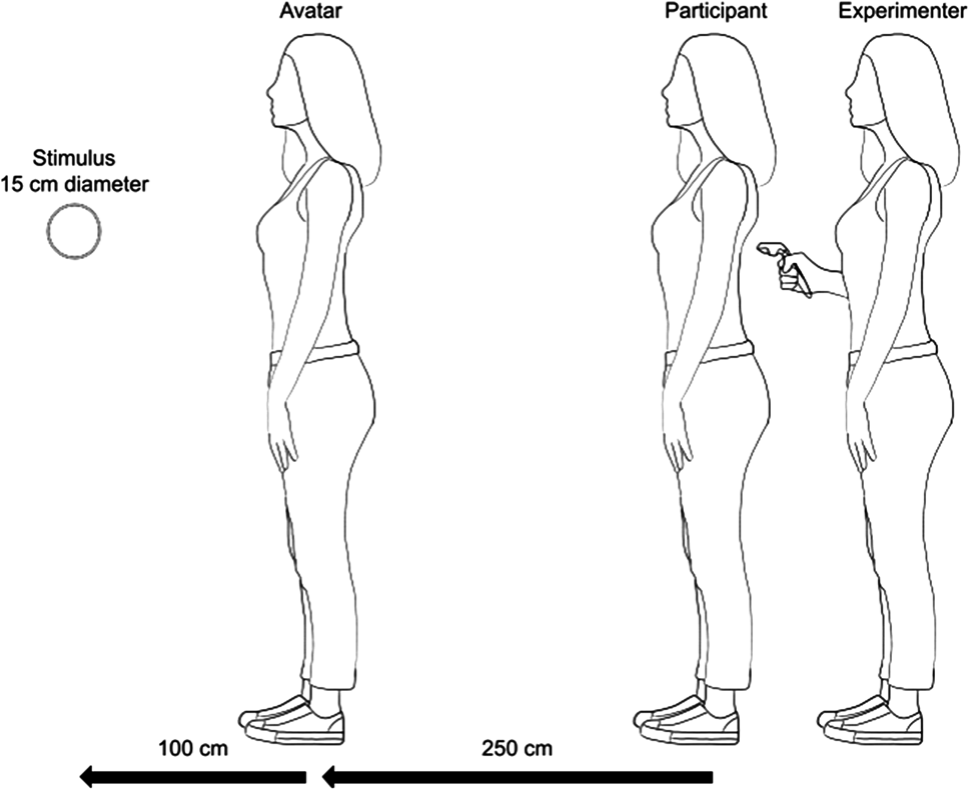
Graphical illustration of the experimental setup. The displayed avatar was only present during the full-body illusion induction periods and was adjusted to the participant’s gender and height. It was positioned 250 cm in front of the participant in the VR environment. The probe stimulus in each trial of the visual depth task was presented 100 cm in front of the avatar and 350 cm in front of the participant.

The full-body illusion was induced by presenting a virtual avatar in front of the participant, while the experimenter stroked the participant’s back. Simultaneously with the physical stroking, the participant saw the virtual avatar being stroked in the head-mounted display^9^. The physical stroking was performed by the experimenter with a tracked hand-held VR controller (see Fig. 6).

**Figure 7:**
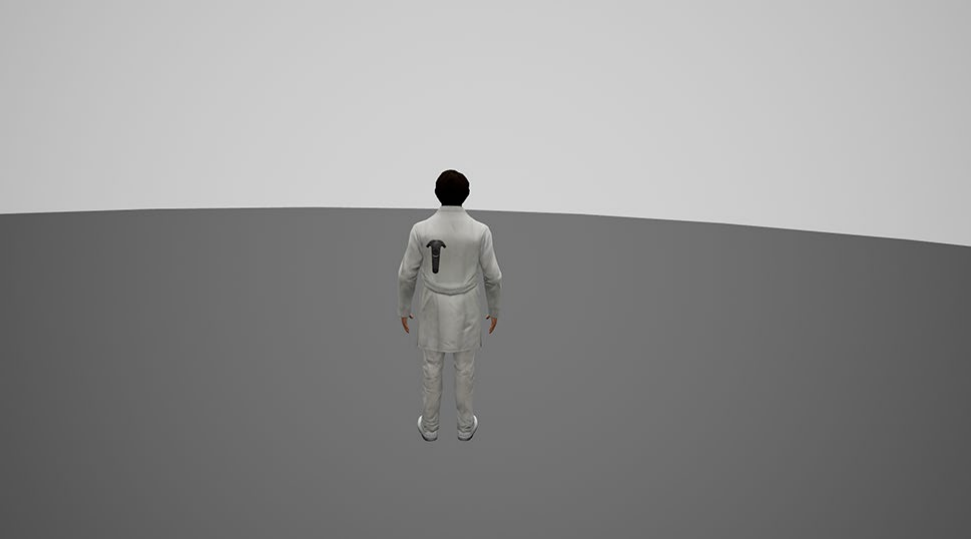
Virtual environment of the experiment during the induction of the full-body illusion from the view point of the participant. The environment only comprised a grey surface to keep potential references to a minimum during the different tasks.

Stimulus presentation was generated by a custom program created with Unreal Engine (version 4.25, https://www.unrealengine.com) and was conducted on a Windows 10 desktop computer (Alienware Aurora R8, Intel Core i7-8700K @3.7GHz, 16 GB RAM, NVIDIA GeForce GTX 1080 graphics card). The virtual environment was run using SteamVR (version 1.17, https://store.steampowered.com/app/250820/SteamVR/) with the SteamVR 1.0 tracking system. Previous research has shown that the system provides suitable tracking of head and hand positions for research purposes if tracking loss is prevented^36^. There were no salient visual reference points in the virtual environment (see Fig. 7).

#### Visual depth task

In the visual depth task participants had to judge the distance of two spheres relative to their perceived self-location in the VR world by indicating which of the two is closer to them with a press on the touchpad of the VR controller. The two spheres had a diameter of 15 cm and were each shown for 200 ms (100 cm apart from each other and 8.13 rotational degree to each side of the central line of sight). The spheres were presented consecutively with an inter stimulus interval of 200–250 ms. We refer to the first presented sphere in a trial as the probe and to the second sphere as the reference. The probe was always presented at a distance of 350 cm in front of the participant, while the position in depth of the reference was systematically varied on the anterior-posterior axis in six equidistant increments (5, 15 or 25 cm further away or closer to the participant), which were presented equiprobably across trials. The side of the first presented sphere and the side of the sphere with the variable position was counterbalanced across trials and randomized in order.

#### Mental imagery task

In the mental imagery task a red ball appeared at the end of the room at an angle of approximately 15° to the left or right from the view direction of the participant^26^. The ball rolled on the floor toward the participant’s viewpoint for 2 s at a constant velocity. Before the ball arrived at the location of the participant, a black screen was displayed. Participants were instructed to imagine the ball continuing just as before and to indicate when the ball would reach the position between their feet by touching the touchpad of the VR controller, which they were holding with their dominant hand.

### Procedure

Experiments utilizing or investigating the full-body illusion usually compare two conditions against each other, one in which the stroking is synchronous to what the participants see on the avatars back and one with asynchronous stroking. The full-body illusion is thought to be successfully induced in the synchronous condition, while the asynchronous one serves as a baseline. In the asynchronous condition, the visual information followed the tactile stimulation, by using a delay between the felt and visual stroking seen in the head-mounted display. We used a delay of 500 ms, which is the most common delay used^26^. Figure 7 shows the virtual environment the participants were experiencing in the head-mounted display during the induction of the full-body illusion. The avatar’s appearance was adjusted and scaled to match the height and the gender of the participant.

The synchronous and asynchronous stroking conditions were tested in separate sessions. The order of all conditions was counterbalanced across participants. During each run the participant performed the visual depth task, the mental imagery task and underwent stroking segments.

During the stroking period, the participant handed the VR controller to the experimenter, who performed a short calibration for the motion tracking. Then, an avatar appeared 250 cm in front of the participant facing straight away. In the following period the stroking was applied to the whole back of the participant, who was instructed to focus on the avatar, which was either stroked synchronously or asynchronously. The duration of this period was 60 seconds in the first block of each individual run and 30 seconds in the following blocks. The avatar was only visible during this period.

At the beginning of a session, the participant performed 10 training trials for the visual depth task and mental imagery task respectively. In the following period participants performed a total of 240 trials of the visual depth task and 40 trials of the mental imagery task split across 20 blocks split into two individual runs. Each block began with the induction of the full-body illusion. Depending on the condition of the respective run, this induction was either synchronous or asynchronous. Afterwards, twelve visual depth task and two mental imagery task trials were performed, which took approximately seven seconds. The order of the two tasks was randomized throughout the whole experiment.

After the last block, an additional stroking period was performed to re-induce the full-body illusion before participants were asked to fill out a questionnaire^22^. The questionnaire was displayed on a Dell Monitor (1920 × 1080 pixel). The questionnaire comprised five different items and participants used a computer mouse to indicate their agreement on a seven-point Likert scale. Item 1 and 3 were chosen to determine changes in perceived self-location. Item 1 inquired participants to what extent they felt as if they were slightly above or below the seen avatar and item 3 to what extent they felt as if their own body shifted toward the seen avatar. Item 2 covered the aspect of self-identification by inquiring the participants to what extent they felt as if the body they saw was their own. Item 4 was intended to quantify the extent of illusory touch experiences by inquiring the participants to what extent the stroking felt as if it was located on the avatar. Item 5 served as a control item and inquired participants to what extent they felt as if nothing changed. Since the original items from Salomon et al.^22^ were in English they were translated into German.

### Statistical analyses

Statistical analyses were performed in python, using the package scipy^37^ (version 1.7.3, https://scipy.org). All reported p-values are Bonferroni corrected when performed in the context of multiple comparisons.

#### Visual depth task

From the participants’ responses in the visual depth task we calculated psychometric functions by fitting cumulative gaussian functions to the average data of each reference sphere position. To estimate discrimination performance in depth, we determined the JND by selecting the variance of the psychometric function. We also determined the PSE, given by the mean of the psychometric function, to estimate the bias in depth perception. The mean number of responses per stimulus level for the computed psychometric functions was 19.62 (SD = 5.17). These calculations were performed for the synchronous and asynchronous condition and for each side of the visual field the probe was presented on individually (left or right visual field), resulting in four values for both, the JND and PSE, for each participant.

#### Mental imagery task

For the mental imagery task, we calculated the distance between the ball and the real position of the participant in the VR world for each trial. These values were then compared between the synchronous and asynchronous sessions.

#### Questionnaire

We estimated the success of inducing the full body illusion by analyzing the subjective measure, i.e. the questionnaire that participants filled out after each session. Since the illusion is elicited when the experimenter strokes the participants’ back synchronously to the stroking seen in the head-mounted display, we compared item scores from sessions with synchronous vs. sessions with asynchronous stroking.

## Data Availability

The datasets analysed during the current study are available from the corresponding author on reasonable request.
